# Reconstructing the west-east genetic division in Indonesia using ancient genomes

**DOI:** 10.1016/j.isci.2026.115974

**Published:** 2026-05-14

**Authors:** Yu Xu, Ketut Wiradnyana, Hui Zhou, Yinhui Zhao, Xian Wang, Le Tao, Kongyang Zhu, Taufiqurrahman Setiawan, Jianhua Wang, Wen-Jing Lu, Yun Wu, Xueping Ji, Chuan-Chao Wang, Xiaoming Zhang

**Affiliations:** 1 State Key Laboratory of Genetic Evolution and Animal Models, Kunming Institute of Zoology, Chinese Academy of Sciences, Kunming, China; 2 Research Center for Prehistoric Archeology and History, National Research and Innovation Agency, Jakarta Pusat, Indonesia; 3 School of Life Sciences, Xiamen University, Xiamen, China; 4 Kunming Natural History Museum of Zoology, Kunming Institute of Zoology, Chinese Academy of Sciences, Kunming, China; 5 Yunnan Key Laboratory of Integrative Anthropology, Kunming Institute of Zoology, Chinese Academy of Sciences, Kunming, China; 6 Yunnan Institute of Cultural Relics and Archaeology, Kunming, China; 7 Research Center for Archaeometry, National Research and Innovation Agency, Cibinong, Bogor, West Java, Indonesia; 8 Institute of Anthropology, Fujian Provincial Key Laboratory of Philosophy and Social Sciences in Bioanthropology, Xiamen University, Xiamen, China; 9 Kunming College of Life Science, University of Chinese Academy of Sciences, Beijing, China; 10 Ministry of Education Key Laboratory of Contemporary Anthropology, Center for Evolutionary Biology, Department of Anthropology and Human Genetics, School of Life Sciences, Fudan University, Shanghai, China; 11 Department of Anthropology, School of Sociology, Yunnan Minzu University, Kunming, China; 12 Guest Student in Kunming Institute of Zoology, Chinese Academy of Sciences, Kunming, China

**Keywords:** Population, Genetics, Genomics

## Abstract

The deep-water Wallace’s line marks a major biogeographic boundary separating western and eastern Indonesia, yet the origin of this genetic divide remains unresolved due to limited ancient genomic data from western Indonesia. In this study, we report two Late Neolithic genomic data from western Indonesia, integrated with 19 published ancient genomes from Island Southeast Asia (7,000 to 200 BP). Our analyses suggest a dual-phase formation of the west-east genetic structure: an Early Holocene west-east divergence, with western forager-related ancestry closer to Hòabìnhian-associated groups and eastern forager-related ancestry closer to Papuan-related groups; and later Neolithic and post-Neolithic demographic processes, including the Austronesian expansion, additional Mainland Southeast Asian gene flow into western Wallacea, and Papuan-associated back-migration into eastern Wallacea, reinforced and reshaped this earlier structure. We therefore propose a revised demographic model in which repeated Holocene migrations acted on pre-existing regional differences to generate the genetic landscape of present-day Indonesian populations.

## Highlights

• Ancient genomes reveal at least early Holocene west-east genetic divide in Indonesia
• Western groups retained Hòabìnhian-related ancestry after Neolithic admixture
• Austronesian expansion spread widely but did not erase regional differences
• Papuan-related ancestry remained strong in eastern Wallacea through time

## Graphical abstract

**Figure:**
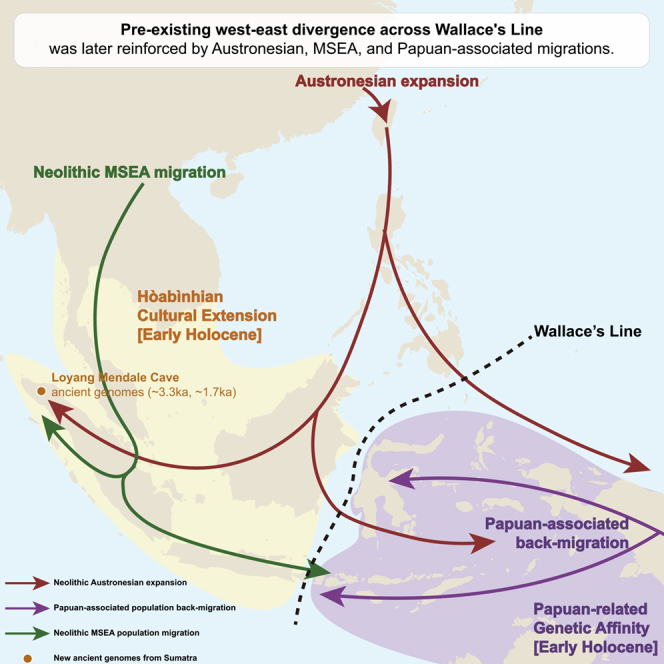

## Introduction

Indonesia, comprising over 17,000 islands, is one of the world’s most genetically diverse and geographically complex regions. Situated at the maritime crossroads between mainland Asia and Oceania, the archipelago is divided by the Wallace line (also known as Wallace’s line). This deep-sea biogeographical boundary demarcates stark differences in biodiversity, ecology, and human history.^1^ During the last glacial maximum, western Indonesia, including Sumatra, Java, and Borneo, formed part of the Sunda Shelf, a contiguous landmass connected to the Asian mainland. In contrast, eastern Indonesia remained separated by deep waters, preserving its ecological and demographic distinctiveness with affinities more closely aligned to Melanesia and the Pacific.^1^

Archaeological evidence indicates that anatomically modern humans (AMH) migrated into Southeast Asia via a southern coastal route, with arrival in both Southeast Asia and Sahul occurring between 65 and 50 thousand years ago.^2^^,^^3^ During the initial dispersal of AMH across Asia, populations rapidly differentiated into at least three major macro-lineages.^4^ Among these, an Australasian lineage associated with present-day Australo-Melanesians and an East/Southeast Asian (ESEA) lineage related to ancient Hòabìnhian hunter-gatherers constitute two ancient branches that have significantly shaped the population structure of Indonesia. Although both groups are deeply divergent from present-day populations in the region, ancient DNA (aDNA) studies have clarified their distinct relationships.^5^ Notably, Hòabìnhian-associated individuals from Laos (7,800 BP) and Malaysia (4,300 BP) show less genetic affinity to Papuan-related groups than to Andamanese.^5^ However, a Toalean-related hunter-gatherer (7,300 BP) from Sulawesi shares most genetic drift and morphological similarities with the Australasian branch,^6^ suggesting a west-east population bifurcation across the Wallace line that was already present in the early Holocene.

Archaeological evidence supports this west-east divide. The Hòabìnhian cultural complex extended from Mainland Southeast Asia (MSEA) into western Indonesia by as early as 12,000 BP,^7^^,^^8^ and ∼8,000 BP with characteristic Sumatralithic tools found at sites such as Loyang Mendale cave in northern Sumatra.^9^ In contrast, eastern Indonesia lacked Hòabìnhian material culture and instead preserves a distinct techno-cultural tradition exemplified by the Toalean technocomplex in Sulawesi.^6^ These records indicate that western and eastern Indonesia were not only ecologically distinct but also demographically and culturally divergent in the Holocene.

This long-standing isolation was transformed by the Neolithization of Islands Southeast Asia (ISEA), particularly through the Austronesian expansion beginning ∼4,000–5,000 years ago.^5^^,^^10^^,^^11^^,^^12^^,^^13^ This movement, originating from mainland southeast China and Taiwan, introduced rice agriculture, red-slipped pottery, and seafaring technologies to both western and eastern Indonesia, leaving a deep imprint on regional material culture.^9^^,^^14^ Parallel to this, Neolithic groups from MSEA migrated into western Indonesia during the same period or slightly later.^13^^,^^15^^,^^16^^,^^17^ These population movements, layered upon existing geographic barriers and cultural divisions, are thought to have played a central role in shaping the complex population structure observed in modern Indonesian groups.

Contemporary genomic studies have consistently reported a pronounced west-east genetic cline across Indonesia, closely aligned with the Wallace line.^18^^,^^19^^,^^20^ All present-day populations carry Austronesian-related ancestry, yet western Indonesians (e.g., Java, Sumatra) are predominantly East Asian-derived, whereas eastern populations (e.g., Timor, Moluccas, Flores) harbor significant Papuan-related components.^20^^,^^21^^,^^22^ This divergence is also reflected in uniparental markers: Y chromosome haplogroups (e.g., O-M122 in the west versus M-P256 in the east) and mitochondrial haplogroups (e.g., B4a1a present in both regions) suggest sex-biased admixture across the archipelago.^19^^,^^20^^,^^23^^,^^24^ Linguistic evidence further mirrors this pattern, with over 700 extant languages divided between Austronesian-speaking groups (predominant across the region) and Papuan-related languages localized in eastern Indonesia.^24^

Recent studies have added new complexity to this demographic picture. Genomic analyses suggest that the Papuan-related ancestry observed in Wallacea may be at least partially attributed to back-migrations from New Guinea beginning around ∼3,500 years ago.^25^^,^^26^ This post-Austronesian Papuan gene flow adds a bidirectional layer to the population history of eastern Indonesia. However, a major limitation in interpreting these patterns has been the scarcity of aDNA from western Indonesia, with only two low-coverage ancient genomes available prior to this study.^5^ As a result, the temporal origins and mechanisms underlying the west-east genetic division remain poorly resolved. Here, we therefore focus on questions that can be tested with Neolithic-to-recent genomes: (1) whether the west-east structure is already detectable by ∼3,300 BP; (2) how Austronesian-related and Mainland Southeast Asian-related ancestries contributed to western Indonesia through time; and (3) how Papuan-related ancestry varied across eastern Indonesia during and after Austronesian-related dispersal.

To address these long-standing questions, we generated and analyzed genome-wide capture data from two ancient individuals excavated at Loyang Mendale Cave in northern Sumatra.^9^ These individuals, dated to 3,347 BP and 1,720 BP respectively, span a crucial demographic transition period in western Indonesia. By integrating these new data with 19 ancient genomes from across ISEA, along with archaeological context and comparative analyses of modern populations, we aimed to reconstruct the spatiotemporal dynamics of human migration, admixture, and population structure across the archipelago.

Loyang Mendale cave offers an ideal context for this investigation. Stratified cultural deposits at the site capture sequential occupations associated with Hòabìnhian, Austronesian, and MSEA Neolithic cultural layers.^9^^,^^16^^,^^17^ The earliest layers (∼8,430–5,040 BP) contain lithic tools and mollusk remains typical of Hòabìnhian sites. The middle phase (∼4,980–1,740 BP) is associated with Austronesian-style polished adzes, ornaments, and ceramics, while the uppermost deposits (around post-2,330 BP) include Sa Huynh-Kalanay pottery and west-east-oriented burials, reflecting cultural transformation or potential population turnover.^9^

By integrating archaeological, genomic, and uniparental evidence from this multi-phase site and placing it within a broader regional framework, we seek to elucidate how Indonesia’s unique geography and long history of human occupation gave rise to one of the most complex genetic landscapes in Southeast Asia. Our findings provide new insights into the origins, persistence, and reshaping of the west-east genetic cline in Indonesia, one shaped not solely by deep-time isolation but also by repeated pulses of migration, local continuity, and sociocultural dynamics across millennia.

## Results

### Two late Neolithic genomes from western Indonesia

In addressing the critical gaps in ancient genomic data from western Indonesia, we screened four skeletal samples from three archaeological sites: Loyang Mendale cave, Putri Pukes cave, and Pangkalan Kitchen Midden site. Of these, only two individuals, designated LMCM1 and LMCM2, exhibited moderate preservation of ancient DNA (aDNA) (Table S1A). The two samples were excavated from Loyang Mendale Cave in Sumatra and directly radiocarbon-dated to 1,720 BP and 3,347 BP, respectively (seen in the method section). This period corresponds to a pivotal phase of demographic transformation in the region, likely shaped by extensive population movements whose impacts are traceable through genomic evidence.

Given the notoriously poor preservation conditions for aDNA in tropical Southeast Asia, attributable to consistently high humidity and temperature,^5^^,^^12^ we employed enhanced molecular methods to improve genome recovery. Specifically, we conducted multiple rounds of single-stranded hybridization capture using the in-solution enrichment protocol targeting over 1 million genome-wide SNPs, as described by Rohland et al.^27^ This approach yielded eight and four single-stranded hybridization capture libraries for LMCM1 and LMCM2, respectively (Table S1A).

We implemented rigorous quality control measures to authenticate ancient origin and minimize modern contamination. Libraries were retained if they met the following criteria: (1) evidence of characteristic postmortem DNA damage (C→T substitution frequency >0.06); (2) recovery of more than 30,000 targeted SNPs on the 1,240 K panel; (3) consistent genetic sex determination using three independent methods; and (4) contamination estimates below 3.5%, assessed via mitochondrial DNA and X chromosome analysis in males. Based on these thresholds, we retained three of eight libraries for LMCM1 and two of four for LMCM2 (Table S1A).

For libraries with >10,000 SNPs but potential contamination, we applied additional filtering to retain only fragments exhibiting authentic damage patterns indicative of ancient DNA (Table S1B). After merging libraries per individual, we recovered two ancient genomes with 171,570 SNPs for LMCM1 and 551,065 SNPs for LMCM2. Both genomes satisfied all authentication criteria (Table S1C and Figure S1A).

Uniparental marker analyses further illuminated the ancestral affiliations of these individuals. Both LMCM1 and LMCM2 carried the Y chromosome haplogroup O1a2, a lineage widely distributed in Southeast Asia and found among indigenous groups in Taiwan.^28^ LMCM1 also carried mitochondrial haplogroup Y2a1, which is similarly prevalent among Southeast Asian populations and Austronesian-speaking communities.^29^ However, mitochondrial haplogroup determination for LMCM2 was inconclusive due to insufficient mitochondrial genome coverage and is thus not reported here.

**Figure 1:**
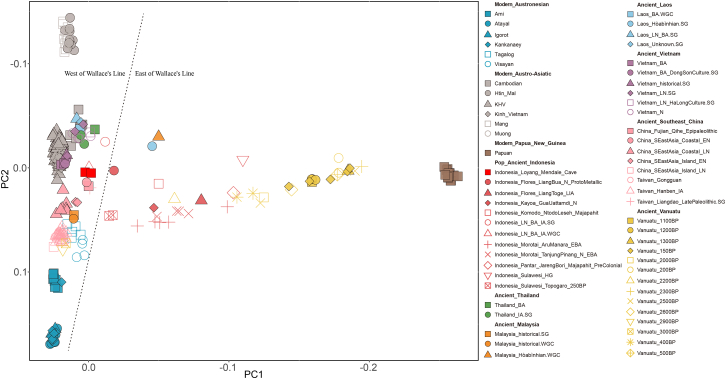
Population structure of ancient indonesian and comparative southeast asian populations/individuals Principal-component analysis (PCA) of ancient populations from Southeast Asia alongside contemporary populations from Papua New Guinea, as well as modern Austronesian- and Austroasiatic-speaking groups. The analysis indicates a robust west-east Wallacean genetic division, with the notable exception of two Hòabìnhian hunter-gatherer individuals (Laos_Hòabìnhian and Malaysia_Hòabìnhian), which cluster with populations east of the Wallace Line. This deviation is likely attributable to the analytical approach: principal components were computed using present-day populations, and ancient individuals were subsequently projected onto this space. The Hòabìnhian individuals, possessing deeper “basal” Southeast Asian ancestry, may therefore exhibit a displacement in PCA space, reflecting their divergent genetic legacy rather than recent admixture.

To investigate the genetic relationships between LMCM1 and LMCM2, we conducted principal component analysis (*PCA*), outgroup*-f*_*3*_ *statistics*, and *qpWave* modeling. All analyses consistently supported a close genetic affinity between the two individuals. Specifically, they occupied overlapping positions in *PCA* space (Figure 1), showed reciprocal best hits in outgroup-*f*_*3*_ analyses (Figure S1B), and displayed genetic homogeneity in *qpWave* results (Rank 0: *p* > 0.05). Given these concordant findings, we grouped LMCM1 and LMCM2 into a single population unit for subsequent population genetic analyses, which we refer to as Indonesia_Loyang_Mendale_cave.

This newly defined population contributes critical genomic data from a previously undersampled region and time period. It provides a robust anchor point for reconstructing demographic transitions in western Indonesia and contributes to broader efforts in mapping population dynamics across ISEA.

### Deep west-east genetic division in Indonesia is unlikely to be explained solely by recent gene flow

A pronounced west-east genetic division characterizes the Indonesian archipelago, a pattern long recognized through modern genomic and linguistic data.^1^ However, the temporal origins of this division remain unresolved due to limited comparative analyses of ancient DNA, raising the question of whether this structure results from recent gene flow or was already established in ancient populations.

To address this, we conducted *PCA* of ancient Indonesian genomes to explore overall population structure (Figure 1). The resulting triangular distribution aligned with major regional ancestries: Austroasiatic, Austronesian, and Papuan. PC1 separated Papuan from Austroasiatic- and Austronesian-speaking populations, while PC2 differentiated Austroasiatic from Austronesian lineages. Strikingly, both PC1 and PC2 partitioned ancient Indonesian populations into two distinct west-east clusters consistent with geographic origin. Western populations clustered toward the left (PC1) and top (PC2), whereas eastern populations exhibited opposite patterns. This observed structure was substantiated by formal statistical correlations: PC1 negatively correlated with longitude (r = −0.61, *p* < 0.05), and PC2 positively correlated with longitude (r = 0.81, *p* < 0.05), while neither axis correlated significantly with latitude (*p* > 0.05) (Figures S2, Figure S3 and Table S1D). These results collectively indicated a robust west-east divergence without north-south stratification in ancient Indonesian populations. However, this interpretation should be treated with caution, as it reflected only the specific sample set and PCA panel used here. Therefore, these findings represented a preliminary observation, and the detailed population history required further investigation through quantitative ancestry modeling.

In ancient DNA research, ancient individuals are often projected onto principal components derived from modern reference populations. The composition of this reference panel can substantially influence the resulting coordinate space, particularly when certain groups, such as Papuan or East Asian populations, drive major axes of variation. To assess the impact of this effect, we explicitly tested how changes in panel composition influenced the inferred structure. Specifically, we repeated the PCA using alternative reference panels without Papuan populations, while keeping the same set of ancient Indonesian individuals as projected samples. We observed that the pronounced west-east genetic divergence was most pronounced when Papuan references were included, reflecting a strong differential affinity for Papuan ancestry between western and eastern Indonesian groups. When Papuan samples were excluded, the separation between western and eastern clusters became considerably less distinct (Figure S6). These comparative results confirm that the observed pattern is partly contingent on reference composition and that PCA alone should be interpreted as descriptive rather than conclusive. It motivates formal tests using *f-statistics* and admixture modeling.

**Figure 2:**
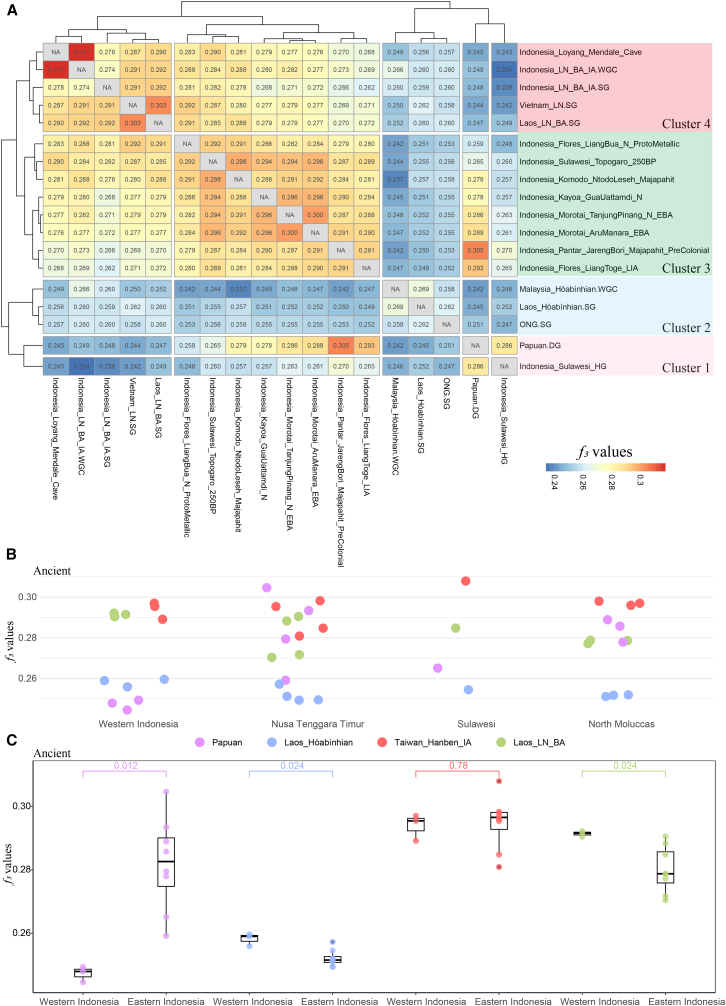
Quantitative *f3*-statistics analyses of ancient Indonesian populations (A) Cluster-based outgroup*-f*_*3*_ analysis assessing shared genetic drift between ancient Indonesian individuals and diverse reference populations. (B) Dot plot displaying the distribution of outgroup*-f*_*3*_ values across distinct Indonesian regions, reflecting regional patterns of genetic affinity. (C) Comparative analysis of genetic affinities in western versus eastern Indonesia with selected reference populations: Papuan, Laos_Hòabìnhian, Taiwan_Hanben_IA, and Laos_LN_BA. Statistical significance was evaluated using the Wilcoxon rank-sum test.

To validate this finding and account for potential PCA bias, we employed hierarchical clustering of pairwise outgroup-*f*_*3*_ statistics of the form *f*_*3*_(*X*, *Y*; Mbuti), where *X* and *Y* are ancient Southeast Asian populations, Onge and Papuan. This analysis yielded four coherent clusters: (1) Papuan-related populations (cluster 1), (2) Hòabìnhian-related hunter-gatherers (cluster 2), (3) ancient eastern Indonesians (cluster 3), and (4) ancient western Indonesians (cluster 4) (Figure 2A). Notably, Hòabìnhian- and Papuan-related groups formed one supercluster (cluster 1_2), while western and eastern Indonesians formed another (cluster 3_4). These patterns indicate that ancient western and eastern Indonesians share more genetic drift within each region than across the Wallace line, and they suggest at least two layers of differentiation: a deep divergence between Hòabìnhian- and Papuan-related lineages, followed by additional Holocene structuring within Indonesia (Figure 2A).

We extended this analysis to modern Indonesian populations. When included in cluster-based *f*_*3*_ analyses, modern groups clustered with either Neolithic/post-Neolithic ancient western or eastern populations, respectively (Figure S2), indicating continuity between ancient and contemporary population structures. These results suggest that the west-east genetic division was likely established at least in the early Holocene and reinforced by later demographic processes. Thus, recent gene flow alone cannot account for the observed structure.

### Basal divergences underpin Neolithic genetic differentiation

To examine the ancestral sources of this division, we re-evaluated the PCA axes: PC1 (Papuan ancestry) correlated negatively with longitude, whereas PC2 (Austroasiatic/Austronesian ancestry) correlated positively (Figure S3 and Table S1D). Together, these correlations are consistent with a west-east gradient in which eastern Indonesians share more Papuan ancestry, whereas western groups are more closely affiliated with MSEA. Besides, these results highlight a predominant west-east genetic differentiation among ancient Indonesian populations. However, the absence of north-south stratification in this PCA should be interpreted with caution, as it reflects only the specific sample composition and single-nucleotide polymorphism (SNP) panel used here. Previous studies, including Oliveira et al. (2023),^13^ have demonstrated additional north-south ancestry distinctions within eastern Indonesia that are not captured in the present analysis.

Outgroup-*f*_*3*_ tests of the form *f*_*3*_(MSEA/Pacific/Austronesian, ancient Indonesians; Mbuti) revealed significant asymmetries. Western Indonesians exhibited greater genetic affinity to MSEA groups, including Hòabìnhian and Neolithic populations (*p* < 0.05, Wilcoxon test), whereas eastern Indonesians showed stronger affinity to Pacific populations (*p* < 0.05) (Figures 2B and 2C). Notably, both groups exhibited comparable levels of drift to Austronesian-speaking groups (*p* > 0.05), suggesting a homogenizing effect of the Austronesian expansion. We note that the absolute *outgroup-f*_*3*_ values span a wide range (Figure 2B), with eastern Indonesian individuals who carry substantial Papuan-related ancestry showing values near ∼0.30, whereas western Wallacean groups show much smaller differences among reference populations. This pattern indicates that, although Hòabìnhian-associated genomes are the best available proxy for the deepest western affinity in our panel, the corresponding signals in western Wallacea are comparatively subtle and should be interpreted as weak affinities to imperfect proxies rather than as direct evidence of continuity from local pre-Neolithic foragers.

**Figure 3:**
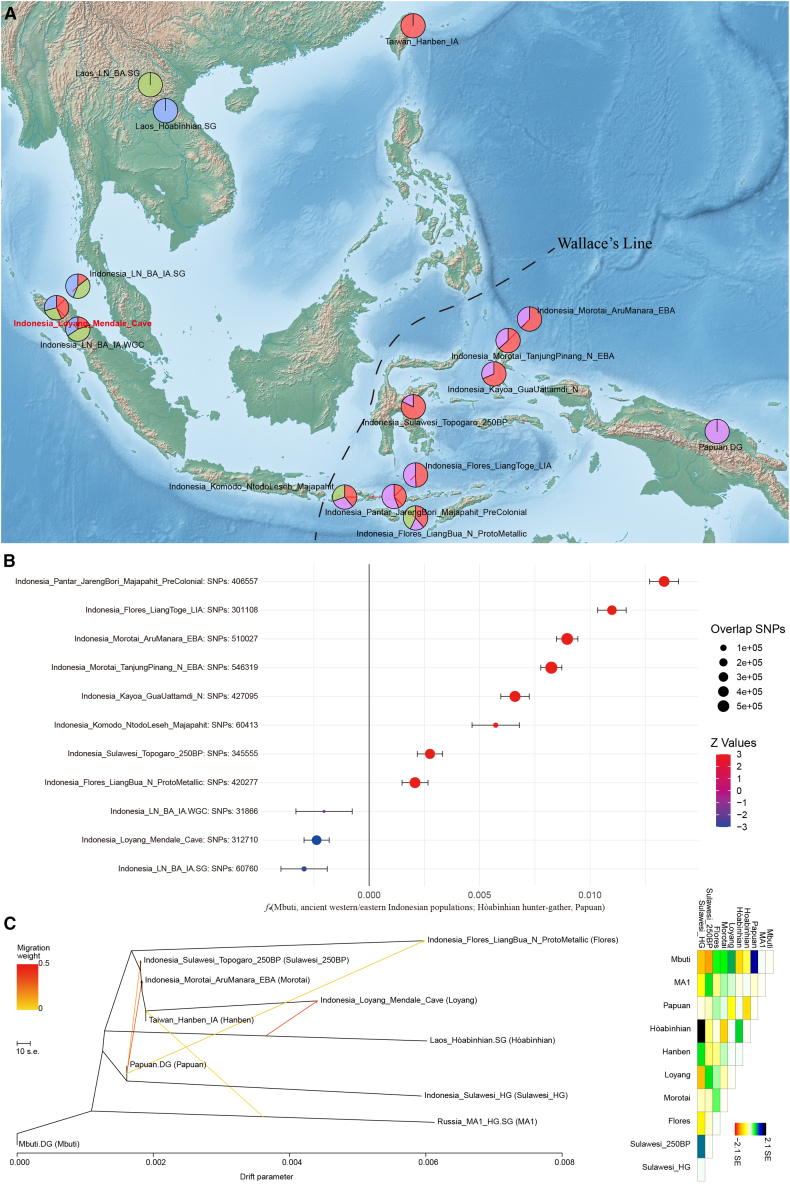
Distinct west-east genetic structure among ancient Indonesian populations (A) Results of *qpAdm* modeling illustrate divergent ancestral compositions between ancient western and eastern Indonesian groups. Base map created using Natural Earth (free vector and raster map data, © naturalearthdata.com). (B) Results of *f*_*4*_ statistics in the form *f*_*4*_(Mbuti, ancient western/eastern Indonesian populations; Hòabìnhian hunter-gatherer, Papuan), highlighting differential affinities to Hòabìnhian and Papuan ancestries. Data are represented as *f*_*4*_values +/− standard errors. (C) *TreeMix* analysis of major ancient Southeast Asian populations with five migration edges, inferred using the set of SNPs overlapping across the included populations (as implemented by *TreeMix*). Migration edges indicate potential gene flow events, and branch lengths reflect genetic drift.

To model ancestry more formally, we applied *qpAdm*. Across the set of plausible models, all ancient Indonesian groups are best fit only when an Austronesian-related source is included. In these models, western groups require an additional Hòabìnhian-related component, whereas eastern groups require a Papuan-related component (Figure 3A and Table S2). These results were further supported by *f*_*4*_*-*statistics of the form *f*_*4*_(Mbuti, ancient West/East Indonesian; Hòabìnhian, Papuan), showing significant positive values (Z > 3) for eastern populations and negative values for western populations (Figure 3B). Only our newly sequenced best-covered western Indonesian genome reached statistical significance (Z = −4.172), underscoring the importance of better-covered genomes for resolving subtle affinities. *TreeMix* analysis inferred multiple migration edges, including three Papuan-related edges into several eastern regions and a Hòabìnhian-related edge into western Indonesia (Figure 3C). Taken together, these results show that Neolithic/post-Neolithic Indonesian population structure is shaped by mixtures involving deeper Hòabìnhian- and Papuan-related divergences, rather than by a single simple replacement event.

### Early Holocene foragers’ genetic legacy, late Neolithic replacement and continuity in modern Indonesian populations

To explore whether ancestries related to early Holocene foragers can be detected in later groups, we included two pre-Neolithic genomes available in the current dataset (Leang Panninge, Toalean technocomplex, Sulawesi; and a Hòabìnhian-associated hunter-gatherer from mainland Southeast Asia).^5^^,^^6^ These individuals form a distinct cluster, genetically distant from Neolithic and later populations in our previous cluster-based outgroup-*f*_*3*_ analyses (Figures 2A and S2), and pairwise mismatch rates highlight substantial differentiation between pre-Neolithic foragers and subsequent populations (Figure S4). Because no pre-Neolithic genomes from western Indonesia are available, we cannot directly test genetic continuity in that region. Instead, using *qpAdm* with the best available proxies, we find that models for later western Indonesian groups frequently require a component associated with Hòabìnhian genomes, whereas models for later eastern Indonesian groups require a Papuan-related component (Figure 3A and Table S2). These estimates depend on proxy choice and model assumptions, and we therefore interpret them as evidence of detectable affinities to deep regional lineages rather than as definitive continuity with local pre-Neolithic populations.

**Figure 4:**
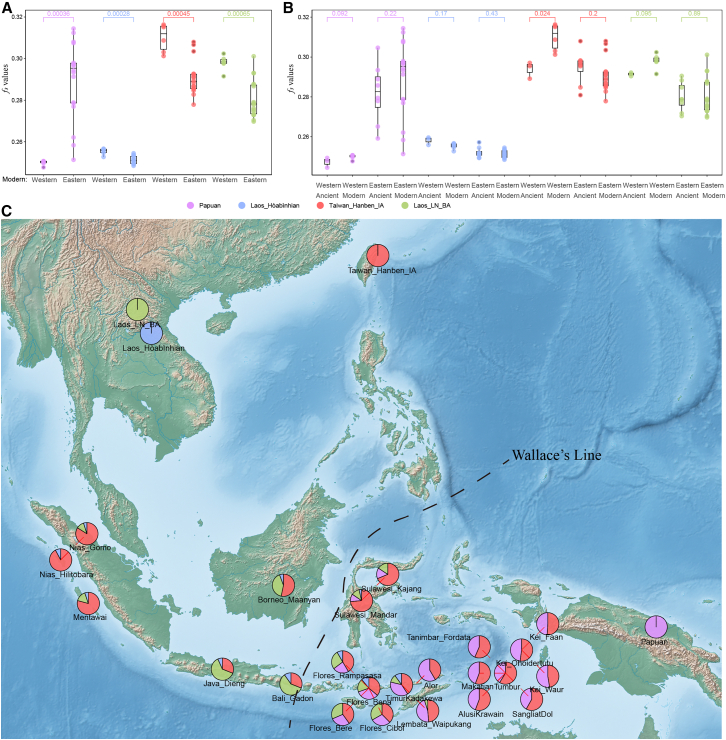
Genetic structure of modern Indonesian populations (A) Outgroup *f*_*3*_*-statistics* comparing modern western and eastern Indonesians with reference populations: Papuan, Laos_Hòabìnhian, Taiwan_Hanben_IA, and Laos_LN_BA, indicate region-specific differences in genetic affinity. Statistical significance was evaluated using the Wilcoxon rank-sum test. (B) Comparisons of outgroup-*f*_*3*_ values between ancient and modern Indonesians indicate varying degrees of genetic continuity and admixture across time. (C) *qpAdm* modeling shows that modern Indonesians harbor mixed ancestries from Papuan-related, ancient MSEA-associated, and Austronesian-derived sources. These results reflect the complex demographic history of the Indonesian archipelago shaped by ancient lineages and multiple migration events.

To assess how these patterns and ancient divergences persist today, we analyzed genomes from present-day living populations using outgroup-*f*_*3*_ and *qpAdm* models. Consistent with Neolithic western and eastern Indonesian populations (Figure 2C), modern western Indonesians also show significantly greater drift with Hòabìnhian and Neolithic MSEA groups, while eastern Indonesians share more drift with Pacific populations (*p* < 0.05, Wilcoxon test) (Figures 4A and S2B). Modern western groups also show stronger Austronesian-related affinities than ancient western groups, consistent with additional post-Neolithic Austronesian-related gene flow in parts of western Indonesia (Figures 4A and 4B).

The *qpAdm* admixture modeling indicated that western populations are best explained as two- or three-way mixtures involving Hòabìnhian (4.9%–7.8%), Neolithic MSEA populations (represented by Laos_LN_BA, 11.2%–62.3%), and Austronesian ancestry (31%–92.2%) (Figure 4C and Table S3). Eastern populations, however, require models incorporating up to four ancestry components, including Papuan (10.9%–58.4%), Austronesian (31%–73.6%), Neolithic MSEA (4.8%–59.1%), and Hòabìnhian (3.6%–11%) (Figure 4C). Overall, these models indicate that late Neolithic and later Holocene movements (Austronesian- and Austro-Asiatic-related) strongly shaped Indonesian genetic variation, while ancestries related to earlier foragers remain detectable at lower levels in many present-day groups.

Regionally, westernmost populations display the highest Austronesian ancestry (average 85.1%), while Papuan-related ancestry is absent west of the Wallace Line. In contrast, eastern populations, excluding Bali (geographically west of the Wallace Line but administratively part of Eastern Indonesia), contain a high proportion of Papuan ancestry (Figure 4C). Bali aligns genetically with Java, consistent with stronger western Indonesian affinities. Several easternmost populations show roughly balanced Papuan- and Austronesian-related ancestry in our models, suggesting relatively stable mixture proportions since at least the Neolithic in the available ancient time transect.

Of particular note, in our *qpAdm* analyses, we were unable to successfully model the ancestry of the Early Holocene Toalean-associated Leang Panninge individual (∼7.3 ka) as deriving solely from a Papuan-related source.^6^ This result agrees with previous findings showing that, although the Toalean individual forms a clade with Papuans, she also carries additional deep Asian ancestry components related to Tianyuan and Onge compared to Papuans. Consequently, modeling Neolithic and modern Indonesian groups using the Toalean individual as a source frequently failed to produce models that met our selection criteria (Table S4). This pattern supports the Papuan back-migration model, assuming that the Toalean genome represents the broader Holocene ancestry of eastern Indonesia. However, considering that Toalean hunter-gatherers and Papuans occupy closely related positions in phylogenetic space, we infer that Papuan-related ancestry in Wallacea was already present by the Early Holocene, with subsequent gene flow from New Guinea likely augmenting rather than initiating this component.

## Discussion

In this study, we present the earliest ancient genomic data to date (3,300 and 1,700 BP) from western Indonesia at Loyang Mendale Cave and integrate them with a broad dataset of ancient and modern genomes across ISEA. The data directly show that by ∼3,300 BP western Indonesian individuals already carried substantial Austronesian-related ancestry while retaining affinities to MSEA-related lineages. Across the available ancient time transect, our analyses suggest that the present-day west-east genetic division reflects both a legacy of early Holocene regional divergence and later Holocene admixture processes, rather than being generated solely by very recent gene flow.

**Figure 5:**
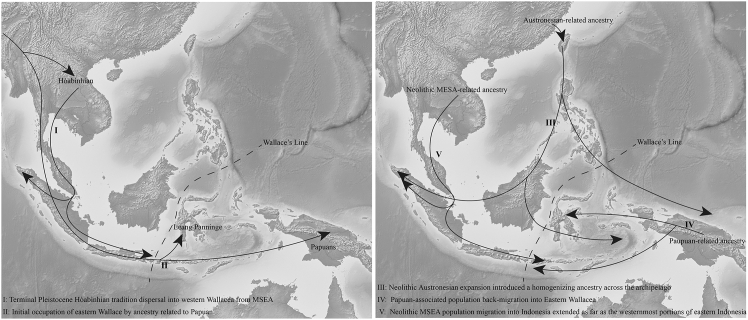
Schematic representation of the proposed two-stage model explaining the emergence of the west-east genomic divergence across the Indonesian archipelago Stage Ⅰ reflects early divergence during the Early Holocene between Hòabìnhian-associated foragers in western Indonesia and Papuan-related Toalean groups in the east, likely shaped by Wallacean biogeographic barriers. Stage Ⅱ involves the Neolithic Austronesian expansion, which introduced widespread East Asian-derived ancestry, yet retained regional heterogeneity through asymmetric admixture, subsequent Mainland Southeast Asian gene flow in the west, and Papuan back-migration in the east.

We propose a two-stage model for the origin of Indonesia’s west-east genetic structure. Stage Ⅰ (suggested by comparative ancient data and archaeological context): a deep divergence at least in the Early Holocene between Hòabìnhian-related foragers in the west and Papuan-related groups (represented by Toalean hunter-gatherer) in the east, likely driven by geographic isolation across the Wallace Line. Stage Ⅱ (directly supported by Neolithic-to-recent genomes): the Neolithic Austronesian expansion introduced widespread East Asian-related ancestry across the archipelago, but did not eliminate regional differences. Instead, asymmetric retention of indigenous components, additional Neolithic/Metal Age MSEA-related gene flow into western Wallacea, and Papuan-associated back-migration into eastern Wallacea,^26^ reinforced and reshaped a longitudinal population structure visible in both ancient and modern genomes (Figure 5). Consistent with this framework, both Neolithic and modern eastern Wallacean populations harbor a very high level of Papuan-related ancestry (Figures 3A and 4C), and they have an equivalent genetic affinity degree with the Papuan population (Figure 4B), suggesting substantial Papuan-related gene flow into the eastern Wallacean lineages during and/or after Austronesian-related dispersals.^26^

However, it is necessary to clarify that our model was constructed by integrating multidisciplinary evidence and was subject to a series of demographic assumptions. For stage Ⅰ of the model, we relied primarily on archaeological and paleogenomic data. Archaeological sites associated with the Hòabìnhian culture expanded from MSEA into western Indonesia around 10,000 BP,^7^^,^^8^ but did not reach eastern Indonesia. Given the current absence of Hòabìnhian-related hunter-gatherer genomes from western Indonesia, we used published Hòabìnhian genomes from MSEA as proxies to compare with the earliest eastern Indonesian hunter-gatherers. Our results showed that these two groups of hunter-gatherers were highly divergent, forming a distinct genetic cluster (Figure 2A) and phylogenetic clade (Figure 3C). Specifically, Hòabìnhian-associated individuals exhibited closer genetic affinity to modern Andamanese populations, whereas mid-Holocene hunter-gatherers from Wallacea^26^ showed closer genetic affinities with Papuans. In addition, we found eastern Indonesian individuals with Papuan ancestry display high *f*_*3*_ values (up to 0.30), whereas western Wallacean individuals show only weak affinities with Hòabìnhian-associated groups (Figure 2). The contrast between the high Papuan-related affinities in the east and the relatively low affinities between western Indonesian populations and Hòabìnhian-related groups suggests that the indigenous forager ancestry in western Indonesia may have been more regionally distinct. Therefore, Hòabìnhian-related groups from MSEA might be represented by the best available proxy at present, rather than a close or definitive source population. Obtaining pre-Neolithic genomes from western Indonesia will be essential to directly evaluate the first stage of our proposed model. Stage Ⅱ of this model was retrieved from time-series genomic data across the archipelago. However, the direct transition process remained unresolved due to a lack of ancient genomes during the key contact period.

A critical remaining question concerns the early local ancestry signals, particularly in eastern Indonesia. It remained unclear whether the Papuan-related signals in later Neolithic populations reflected a persistent Early Holocene ancestry or were driven by a distinct Papuan back-migration. Our analyses indicated that mid-Holocene hunter-gatherers from Wallacea were less closely related to the genetic components found in Neolithic and post-Neolithic eastern populations, as suggested by *qpAdm* modeling (Figures 3A and 4C) and *TreeMix* analysis (Figure 3C). Nevertheless, considering that Toalean hunter-gatherers and Papuans occupied closely related positions in phylogenetic space (Figure 3C), we proposed that Papuan-related ancestry in Wallacea was likely already present by the Early Holocene, with subsequent gene flow from New Guinea augmenting rather than initiating this component. Consequently, broader and earlier genomic sampling in eastern Indonesia is required to provide a definitive test of this stage.

Integrating genomic results with archaeological evidence for widespread pre-Neolithic foraging traditions (including Hòabìnhian and Toalean technocomplexes), our analyses suggest that ancestries related to early Holocene foragers contributed to later populations. At the same time, pre-Neolithic individuals are deeply divergent from many post-3,000 BP groups in our *f-statistic* clustering and *qpAdm* comparisons (Figures 2A, 3A, and S5). Together, these observations support a scenario of both ancestry persistence and substantial demographic change through the Neolithic and later periods.

Our *qpAdm* modeling recovers distinct ancestry components among Neolithic western Indonesians: a tripartite mixture involving Taiwan_Hanben_IA-related (43.3%), Laos_Hòabìnhian-related (29.1%), and Laos_LN_BA-related (27.6%) sources (Figure 3A and Table S2). These proportions are model-dependent and should be interpreted as estimates under the chosen proxy references, but they consistently indicate multiple mainland- and Taiwan-related contributions in western Indonesia. This genetic profile complements the archaeological record from Loyang Mendale cave, which documents successive occupations associated with Hòabìnhian foragers, Austronesian expansions, and MSEA cultural influences. The concordance between stratified cultural phases and distinct ancestry components suggests that these material culture transitions were accompanied by episodes of human mobility and admixture rather than by cultural transmission alone. Notably, this genetic pattern was not unique to our newly sequenced samples; two previously reported low-coverage genomes from western Indonesia^5^ showed the same tripartite structure, providing robust evidence for broader demic and cultural co-diffusion across western Indonesia. In contrast, several easternmost populations are well fit by simpler Papuan-related and Austronesian-related mixtures, and some populations near the transitional zone (e.g., Flores) show additional MSEA-related signals (Figure 4C). Discrepancies with previous studies (e.g., Oliveira et al.^13^) likely reflect differences in reference panels, sample composition, and statistical power, including variation between 1,240K and HO-based *qpAdm* runs (Table S2D). Overall, the available models suggest regionally limited eastward movement of some MSEA-related ancestry beyond the Wallace line during the Neolithic/Metal age.

Our results are also consistent with the Papuan back-migration model proposed in recent studies,^25^^,^^26^ but they also allow a complementary interpretation. Because Toalean and Papuan references occupy closely related phylogenetic positions, the Papuan-related ancestry in later eastern Indonesian groups could reflect both persistence of Papuan-related ancestry already present by the Early Holocene and additional later gene flow from New Guinea. Our data do not uniquely identify the timing of all Papuan-related inputs; denser temporal sampling in eastern Indonesia will be required to more definitively distinguish continuity from later pulses. Finally, in our modeling framework, Onge-related ancestry shows stronger affinity to Hòabìnhian-associated hunter-gatherers than to Papuan references, suggesting it may track an MSEA-specific deep Asian branch rather than a universal founder component.

Our data show that western Indonesia was already integrated into the Austronesian expansion zone by around 3,300 BP, corroborated by archaeological evidence of rice agriculture, red-slip pottery, and maritime networks.^30^ In addition, the Iron Age Austronesian population from Taiwan (Hanben_IA) shows a significantly higher proportion of drift alleles among modern populations than among Late Neolithic populations in western Wallacea (Figure 4B), consistent with additional post-Neolithic Austronesian-related admixture in parts of western Indonesia. Although the two Loyang Mendale individuals carry uniparental markers common in Austronesian-speaking populations, Y chromosome lineages in parts of eastern Indonesia show increased frequencies of Papuan-associated haplogroups (e.g., M-P256 and C1b2a1a), while mitochondrial lineages often remain East Asian-derived (e.g., B4a1a1 and F3b).^24^ This sex-asymmetric pattern is consistent with male-biased Papuan-related admixture, matrilocality and ethnographic observations across Oceania,^12^^,^^20^^,^^23^^,^^31^ though the underlying social processes (e.g., matrilocality) remain interpretations that should be tested with additional uniparental and isotopic data.

### Conclusions and future directions

The genomic structure of the Indonesian archipelago reflects a complex interplay of deep-time divergences and more recent demographic processes. A persistent west-east genetic division, evident in both ancient and modern populations, is underpinned by asymmetric ancestry contributions: Hòabìnhian-related lineages in the west and Papuan ancestry in the east. The Austronesian expansion overlaid a widespread East Asian-related ancestry onto a deeper, pre-existing genetic division across the Wallace line. However, it did not entirely erase this earlier ancestry, and the pre-Neolithic west-east structure persists in present-day populations.

Our new ancient genomes from Western Indonesia improve temporal and geographic coverage for testing models of Indonesian population history. Combined with published ancient time series, the results support a picture of mixed continuity and turnover: detectable contributions from early Holocene-related ancestries, substantial Holocene admixture associated with Austronesian- and MSEA-related movements, and regionally variable Papuan-related gene flow in the east. We interpret uniparental patterns and autosomal admixture gradients as being consistent with sex-biased processes in parts of Wallacea, while emphasizing that additional sampling will be needed to quantify these dynamics robustly. Overall, Wallacea emerges as a long-term contact zone in which geography constrained movement but did not prevent repeated interaction between Asia- and Oceania-related lineages. These findings advance our understanding of the genetic formation of one of the most linguistically and culturally diverse regions on Earth.

Moving forward, a finer-scale, more temporally resolved genomic dataset, especially from transitional and under-sampled regions and from Late Pleistocene fossils, will be essential to elucidate the complex peopling of ISEA. These findings carry broader implications for understanding how geography, culture, and biology intersect to produce enduring patterns of human diversity in maritime Southeast Asia.

### Limitations of the study

Despite providing the earliest direct ancient genomic evidence from western Indonesia, this study is constrained by temporal and geographic gaps in the dataset. Ancient genomes remain sparse, particularly from pre-Neolithic western Indonesia and underrepresented regions such as Borneo, Sulawesi, and the Lesser Sunda Islands. Our key inferences about Early Holocene divergence rely on using Hòabìnhian genomes from MSEA and a single Toalean genome as proxies, because comparable early genomes from western Indonesia are not yet available; this limits our ability to date the stage I split directly. Additionally, panel composition and coverage biases may influence *qpAdm* estimates and model fits; different plausible proxy choices can shift inferred mixture proportions, even when the qualitative pattern (west enriched for Hòabìnhian-related ancestry; east enriched for Papuan-related ancestry) remains stable. *TreeMix* migration edges represent inferred graph features rather than unique historical events and should be interpreted as hypothesis-generating rather than definitive evidence for the number or direction of migrations. Future high-resolution genomic sampling across diverse ecological and cultural contexts will be critical to refining our understanding of intra-island variation, migration routes, and admixture dynamics.

## Resource availability

### Lead contact

Further information and requests for resources and reagents should be directed to and will be fulfilled by the lead contact, Xiaoming Zhang (zhangxiaoming@mail.kiz.ac.cn).

### Materials availability

This study did not generate new unique reagents.

### Data and code availability

• Alignment files (BAM format) and genotype data have been deposited in the Genome Sequence Archive (GSA) in National Genomics Data Center, China National Center for Bioinformation/Beijing Institute of Genomics, Chinese Academy of Sciences. Accession numbers are listed in the key resources table.
• This paper does not report the original code.
• Any additional information required to reanalyze the data reported in this paper is available from the lead contact upon request.

## Acknowledgments

This study was supported by the 10.13039/501100001809National Natural Science Foundation of China (grants T2222030, T2425014, U23A20161, and 32270667), the Yunling Scholar of the Xingdian Talent Support Program (Yunnan Province), the 10.13039/501100003392Natural Science Foundation of Fujian Province of China (2023J06013), and the Major Project of the 10.13039/501100012456National Social Science Foundation of China (21&ZD285). We also acknowledge the 10.13039/501100012166National Key Research and Development Program of China (2024YFC3306701, 2023YFC3303701-02). We sincerely thank the reviewers for their thoughtful and constructive suggestions, which have substantially improved the quality and clarity of this manuscript.

## Author contributions

X.Z., C.-C.W., X.J., and K.W. conceived and designed the study. K.W. led archaeological excavations, K.W., X.J., X.Z., X.W., and Y.W. carried out field investigation and collected the samples. Y.X., H.Z., Y.Z., L.T., and K.Z. performed the data analysis. Y.Z. and X.W. conducted laboratory work on the samples. W.-J.L. participated in laboratory preparation. Y.X., X.Z., C.-C.W., T.S., and J.W. wrote the manuscript. Y.X., X.Z., C.-C.W., and H.Z. revised the manuscript. All authors have read and approved the final version.

## Declaration of interests

The authors declare no competing interests.

## STAR★Methods

### Key resources table

**Table:** 

REAGENT or RESOURCE	SOURCE	IDENTIFIER
**Biological samples**		

Ancient skeletal element	This paper	LMCM1
Ancient skeletal element	This paper	LMCM2

**Chemicals, peptides, and recombinant proteins**		

T4 Polynucleotide Kinase (10 U/uL)	ThermoFisher	EK0032
KAPA HiFi HS Uracil +ready Mix	KAPA Biosystems	KK2802
ATP (100 mM)	Fermentas	R0441
dNTP Mix (25 mM each)	ThermoFisher	R1121
Dynabeads MyOne Streptavidin C1	Invitrogen™	65001
AccuPrime Pfx DNA polymerase	ThermoFisher	12344024
T4 RNA ligase reaction buffer(10x)	NEB	B0216L
FastAP thermosensitive alkaline phosphatase(1U/μL)	ThermoFisher	EF0651
T4 DNA ligase, 5 U/μL	ThermoFisher	EL0012
T4 DNA ligase, high concentrated 30 U/μL	ThermoFisher	EL0013
Klenow fragment (10 U/uL)	ThermoFisher	EP0052
PB buffer	Qiagen	19066
AMPure XP Beads	Beckman	A63882
Isopropyl alcohol	Macklin	I811932-4L
Anhydrous ethanol, pharmaceutical grade, 99.5%	Mreda	M206333-5L
5M NaCl	Sigma-AldriCE	S5150-1L
Nuclease-free water	Thermo Fisher	4387936
20× SSC buffer	Sigma-AldriCE	S6639-1L
3 M Sodium acetate, pH 5.2	Sigma-AldriCE	S7899
Ethanol (Analytical Reagent)	Sigma-AldriCE	459844
20% (wt/vol) SDS solution	Thermo Scientific	J63394.AK
Tris (1 M)、pH 8.0、no RNase	Thermo Scientific	AM9856
Guanidine hydrochloride	Sigma-Aldrich	G3272-2 kg
Water, HPLC grade	Sigma-Aldrich	AH365-4HC
Proteinase K (10 mg/mL)	Sigma-Aldrich	P6556
0.5M EDTA (pH8.0)	Applichen	A48921000
NaClO	Sinopharm	80010428
Agarose	Biowest	111860

**Critical commercial assays**		

Qubit dsDNA Quantitation Kit	Invitrogen	Q32854
MinElute PCR Purification Kit	QIAGEN	28006
Twist Mitochondrial Panel Kit	Twist	102040
Twist Binding and Purification Beads	Twist	100984
Twist Universal Blockers	Twist	100767
Twist Hybridization Reagents	Twist	100982
Twist Wash Buffers	Twist	100846
Twist Ancient Human DNA Panel	Twist	106658

**Deposited data**		

BAM files reported in this paper have been deposited in the GSA-Human (https://ngdc.cncb.ac.cn/gsa-human/).	This paper	GSA-Human: HRA012012
Genotype data reported in this paper have been deposited in the OMIX, (https://ngdc.cncb.ac.cn/omix/).	This paper	OMIX: OMIX010699

**Oligonucleotides**		

Phosphate-AGATCGGAAGAAA[A][A][A] [A][A][A][A]-TEG-Biotin	Gansauge et al.^32^	1st adapter
SpacerC12-[A][A][A]CTTCCGATCTNNNNNNNN[A]-AminoC6	Gansauge et al.^32^	Splinter
GTGACTGGAGTTCAGACGTGTGCTCTTCC∗G∗A∗T∗C∗T	Gansauge et al.^32^	Extension primer
CGACGCTCTTC-ddC	Gansauge et al.^32^	2ND adapter, strand1
Phosphate-GGAAGAGCGTCGTGTAGGGAAAGAGTGTA	Gansauge et al.^32^	2ND adapter, strand2
ACACTCTTTCCCTACACGACGCTCTTCC	Gansauge et al.^32^	Sequencingprimer
Phosphate-ATTCAGCTCCGGTTCCCAA CGATCAAGGCGAGT TACATGA-Phosphate	Gansauge et al.^32^	Control DNA

**Software and algorithms**		

AdapterRemoval v2.3.3	Schubert et al.^33^	https://github.com/MikkelSchubert/adapterremoval; RRID: SCR_011834
BWA v0.7.17	Li et al.^34^	https://bio-bwa.sourceforge.net/; RRID: SCR_010910
SAMtools v1.18	Li et al.^35^	http://samtools.sourceforge.net; RRID: SCR_002105
bamUtil v1.0.15	Jun et al.^36^	https://github.com/statgen/bamUtil
DeDup v0.12.8	Peltzer et al.^37^	https://github.com/apeltzer/DeDup
pileupCaller	https://github.com/stschiff/sequenceTools	https://github.com/stschiff/sequenceTools
PMDtools v0.60	Skoglund et al.^38^	https://github.com/pontussk/PMDtools
ContamMix	Fu et al.^39^	https://github.com/plfjohnson/contamMix
ANGSD v0.940	Korneliussen et al.^40^	http://www.popgen.dk/angsd/index.php/ANGSD; RRID: SCR_021865
HaploGrep2 v2.4.028	Weissensteiner et al.^41^	https://haplogrep.uibk.ac.at/index.html
YLeaf v2.2	Ralf et al.^42^	https://github.com/genid/Yleaf
EIGENSOFT	Patterson et al.^43^	https://github.com/DReichLab/EIG; RRID: SCR_004965
ADMIXTOOLS (*qp3Pop*, *qpDstat*, *qpWave*, *qpAdm*)	Patterson et al.^44^	https://github.com/DReichLab/AdmixTools/; RRID: SCR_018495

### Experimental model and study participant details

#### Sample information

Our study exclusively involves ancient human remains and does not include animals, cell lines, or experimental model organisms.

The study subjects comprise two ancient human individuals (LMCM1 and LMCM2) excavated from Loyang Mendale Cave in Sumatra, Indonesia. Both individuals belong to the species *Homo sapiens* and are dated to the Late Neolithic period, approximately 3,300 BP and 1,700 BP, respectively. Genome-wide genotype data were generated using targeted enrichment of approximately 1.2 million SNPs, and ancestry was inferred through population genetic analyses. These two newly reported individuals were analyzed together with 19 previously published ancient genomes from Island Southeast Asia, resulting in a combined dataset of 21 ancient individuals.

The two newly sequenced individuals were evaluated for genetic similarity using principal component analysis, outgroup *f*_*3*_-statistics, and *qpWave* modeling, all of which consistently indicated genetic homogeneity. Accordingly, they were grouped into a single population unit (Indonesia_Loyang_Mendale_Cave) for downstream analyses. As this study is observational and based on ancient genomic data, no experimental grouping, randomization, or allocation procedures were applied. Instead, individuals were grouped according to genetic affinity, geographic origin, and temporal context for population genetic analyses.

Genetic sex was determined using X- and Y chromosome based methods as described in the Methods section. However, due to the limited sample size (*n* = 2) and the population-level focus of the analyses, it is not possible to assess the influence of sex or gender on the study results for the Loyang Mendale Cave population.

#### Community consultation and ethical consent

All research activities involving ancient human remains were conducted under a collaborative framework that emphasized local engagement, ethical compliance, and cultural sensitivity. Prior to sampling and archaeogenetic analyses of individuals from Loyang Mendale Cave, formal consultation was undertaken with local government and community representatives. Research permits for excavation, sample export, and DNA analysis were issued by the Research Center for Prehistoric Archeology and History, National Research and Innovation Agency, Indonesia, following national regulatory procedures.

In alignment with ethical best practices in archaeogenomic research, the research team engaged in dialogue with the local community before, during, and after the excavation process. These interactions included fostering transparency and allowing the community to observe and comment on excavation and conservation practices. Research findings, including results from DNA analyses, were regularly communicated to community members in an accessible manner to support knowledge-sharing and mutual understanding.

After completion of laboratory analysis, all human remains were respectfully repatriated to the Research Center for Prehistoric Archeology and History, National Research and Innovation Agency of Indonesia. These integrated efforts reflect our commitment to community consultation, institutional transparency, and the co-production of scientific knowledge within a culturally respectful framework.

### Method details

#### Direct AMS dates

In this study, two human bone collagen samples were directly dated using accelerator mass spectrometry (AMS) at Guangzhou Carbon Year Technology Co., Ltd. The resulting radiocarbon data were calibrated using OxCal v4.4.4^45^ and the IntCal 20^46^ calibration curve. All dates are reported in years before present (BP), where “present” is defined as AD 1950. The dating results are as follows:

LMCM1:1,818-1,622 cal BP.

LMCM2:3,442-3,253 cal BP.

#### Ancient DNA laboratory works

Four bone or tooth specimens from 3 archaeological sites were testes in this study. All experiments were conducted in ancient DNA-dedicated laboratory in the Molecular Palaeontology Research Group (MPG) at the Kunming Institute of Zoology, Chinese Academy of Sciences. To eliminate surface contaminants, the samples were treated with 75% ethanol followed by 10% sodium hypochlorite (NaClO). After mechanical abrasion of the outer surface and ultraviolet (UV) irradiation, 50 to150 mg of inner cortical bone powder or tooth powder was obtained using a drill. Ancient DNA was extracted from the resulting bone or tooth powder following a previously published protocol.^47^^,^^48^ To preserve the characteristic terminal damage patterns of ancient DNA, single-stranded libraries were prepared without uracil-DNA glycosylase (UDG) treatment.^32^ Subsequently, we employed a hybridization capture method using Twist Bioscience reagents, following the protocol developed by David Reich’s laboratory, to enrich for approximately 1.2 million SNPs.^27^ The enriched libraries were then sequenced on the MGI-DNBSEQTM-T7 platform (2 × 75 cycles).

#### Ancient DNA sequence data processing

Adapter sequences were trimmed, low-quality reads (quality <20) and short reads (<30 bp) were filtered, and paired-end reads were merged into single sequences using *AdapterRemoval v2.3.3* with the parameters “--minlength 30 --trimns --trimqualities --minquality 20 --collapse”.^33^ The merged reads were mapped to the human reference genome hs37d5 (GRCh37 with decoy sequences) using *BWA v0.7.17*^34^ with the parameters “aln -l 1024 -n 0.01”. PCR duplicates were removed with *DeDup v0.12.8*,^37^ and reads with mapping quality (MAPQ) below 30 were excluded using *SAMtools v1.18*.^35^

To ensure the authenticity of ancient DNA, we used *PMDtools v0.60*^38^ to calculate the ancient DNA damage patterns. Sample contamination was estimated using *ContamMix*^39^ and *ANGSD v0.940*.^40^ Samples exhibiting contamination rates >3.5% underwent additional filtering with *PMDtools v0.60*^38^ (--threshold 3) to retain only fragments displaying characteristic ancient DNA damage patterns.

To account for DNA damage, read ends were trimmed using *BamUtil v1.0.15*,^36^ with transition rates restricted to <3% (or <6% for samples subjected to damage-specific filtering, total cut length no longer than 30). Pseudo-haploid genotypes were generated for the 1240k and HO panels using *pileupCaller v1.5.3* (https://github.com/stschiff/sequenceTools) with the parameters “--randomHaploid -q30 -Q30”.

Genetic sex was inferred by calculating the Rx,^49^ Ry^50^ and the genome coverage between X and Y chromosomes,^51^ with concordant results required for confident assignment. Mitochondrial DNA haplogroups were classified using *HaploGrep2 v2.4.0*.^41^ Based on the high coverage of mitochondrial DNA sequencing data, individual LMCM1 was confidently assigned to haplogroup Y2a1. In contrast, LMCM2 yielded only 821 reads mapped to the mitochondrial genome, providing insufficient depth and breadth at key diagnostic sites to support a reliable haplogroup determination (Table S1).

Y chromosome haplogroups were first determined with *YLeaf v2.2*,^42^ followed by manual verification. Y chromosome haplogroups were determined through the analysis of single-nucleotide polymorphism (SNP) states in accordance with version 15.73 of the ISOGG Y-DNA Haplogroup Tree (https://isogg.org/tree/index.html). The revision dates for each haplogroup classification are as follows:

Sample LMCM1 was classified as haplogroup O1a2 (O-M110), supported by derived SNPs F2671 (18018434G→A), F3134 (21143756G→A), F3214 (21493930T→C), and Y33198 (23590469T→C). Upstream mutations corresponding to haplogroup O (e.g., M1738, M1741, P186, FGC12433, M1747, F315, and others) and O1 (M1297) were also observed. However, the sample retained ancestral alleles for downstream markers of subclade O1a2a1a (B393) and O1a2a2 (e.g., F1600 and F26627), confirming its placement within O1a2 without assignment to more specific terminal branches.

Sample LMCM2 could be tentatively assigned to haplogroup O1a2 (O-M110), based on derived SNPs F2380 (17110058C→G), F2671 (18018434G→A), F3152 (21216583T→C), M50 (21868672T→C), and Y33198 (23590469T→C). Additional upstream mutations (e.g., M1738, CTS2419, F315, F537, M1781, Y14034) supported its placement within O. Nevertheless, due to the limited coverage of the Y chromosome in this individual, the reliability of this assignment remains uncertain, and deeper subclade resolution cannot be confidently achieved.

Haplogroup O and its subclades: revised on 1 October 2019.

#### Data collection

We merged our dataset with the Allen Ancient DNA Resource (AADR) v54.1.p1,^52^ using *mergeit v2450* in *EIGENSOFT*. The merged dataset was subjected to stringent quality filters: (1) retention of only data flagged as “PASS” in column 34 of the.anno file; (2) exclusion of low-coverage individuals (<15,000 SNPs); (3) removal of duplicated or closely related individuals identified within the reference datasets.

In addition, we also merged two different modern datasets for further analysis.^53^^,^^54^

#### Principal component analysis

We conducted principal component analysis (PCA) using smartpca v18140^43^ with the “Human Origin” (HO) dataset to maximize modern population representation. Ancient samples were projected onto PCs calculated from modern populations using the parameters “lsqproject: YES, altnormstyle: NO, and numoutlieriter: 0”.

#### Quantitative analysis

We conducted *f*_*3*_ statistics using *qp3Pop v651*^44^ with the parameter “inbreed: YES”. For *f*_*4*_-statistics, we employed *qpDstat v980*^44^ with parameters “*f4*mode: YES, printsd: YES, inbreed: YES”.

For the hierarchical cluster-based pairwise outgroup-*f*_*3*_ analyses, we first performed outgroup-*f*_*3*_ statistics in the form of *f*_*3*_(Test populations, Test populations; Mbuti), where the Test populations represented the same list of populations. Next, we organized the pairwise *f*_*3*_ results into a matrix and used the *pheatmap* package (https://cran.r-project.org/web/packages/pheatmap/index.html) in R to conduct hierarchical clustering and generate a heatmap. Specifically, the clustering method in *pheatmap* first computes distances based on Euclidean distance, followed by hierarchical clustering using the *hclust* function with the complete linkage method.

Population structure and admixture analyses were performed using *qpWave* v410 and *qpAdm* v810^44^ with the shared parameters “details: YES, allsnps: YES, inbreed: YES”.

For *qpAdm* analysis, the parameter “allsnps” determines how SNPs are selected for *f*_*4*_-statistic comparisons. When “allsnps: NO”, *qpAdm* restricts the analysis to the intersection of SNPs present in all populations, which can be appropriate for analyses of high-coverage samples. However, when ancient samples with substantial missing data are included, setting “allsnps: YES” is generally recommended. With “allsnps: YES”, each *f*_*4*_ statistic is computed using all SNPs available for the four populations involved instead of forcing a single global intersection at the start of the analysis.^55^

*qpAdm* was applied using the rotation method, with sources not included in the admixture models serving as outgroups. Model selection followed several criteria: (1) *p*-values: models with *p*-values >0.01 were considered statistically plausible; (2) admixture proportions and standard errors (SEs): estimated admixture proportions and their SEs were required to fall within the range of 0–1; (3) admixture proportion threshold: the lower bound of each admixture proportion (estimate minus SE) had to be greater than 0. In addition, model evaluation incorporated p-nest values (with 0.01 as the threshold) and *f*_*4*_-statistics to identify the best-fitting models.^56^

#### Phylogenetic tree construction

In our *TreeMix* analysis, we selected three representative ancient eastern Indonesian populations from distinct geographic regions: Indonesia_Morotai_AruManara_EBA (North Moluccas), Indonesia_Flores_LiangBua_N_ProtoMetallic (Nusa Tenggara Timur), and Indonesia_Sulawesi_Topogaro_250BP (Sulawesi). These samples represented the oldest available Neolithic/post-Neolithic individuals from each respective region. For western Indonesia, we included our newly reconstructed genomes. We inferred a phylogenetic tree using *TreeMix v1.13*,^57^ with Mbuti as the outgroup. Using the overlapping SNPs set, Analyses were performed with parameters “-root Mbuti.DG -m -se -bootstrap -k 500 -global -noss” to model different migration events (m). We generated 1,000 bootstrap replicates for each migration scenario and constructed consensus trees using *Phylip v3.697* (consense function).^58^ Final trees were generated by re-running *TreeMix* with the “-tf” parameter on consensus topologies.

### Quantification and statistical analysis

We conducted all statistical analyses using R 4.3.1. All the statistical details of experiments can be found in Results and Figure legends.

## Supplementary information

Document S1. Figures S1–S6

Table S1. Genomic summary statistics and principal component analysis results for newly reported ancient samples This table provides an overview of genomic characteristics and analytical outcomes for the ancient individuals newly reported in this study. (A) Per-library sequencing metrics, including endogenous DNA content, coverage, and duplication rates. (B) Postmortem damage (PMD)-filtered metrics generated using *PMDtools*, reflecting authentic ancient DNA signals. (C) Combined metrics for merged libraries from individuals LMCM1 and LMCM2 to maximize usable genomic data. (D) Principal component analysis (PCA) results for ancient Indonesian samples, including individual coordinates projected onto the reference space. Related to STAR Methods.

Table S2. qpAdm admixture modeling results for ancient Indonesian populations This table presents the results of admixture modeling performed using *qpAdm* to explore ancestry composition in ancient Indonesian individuals. (A–C) Results based on the 1,240K SNP panel: (A) Summary of basic model configurations, including source and outgroup populations. (B) Full list of all tested admixture models, regardless of statistical fit. (C) Subset of models that passed the filtering criteria outlined in the Methods (e.g., *p-*value thresholds, ancestry proportion bounds); models selected for interpretation are highlighted in yellow. (D) *qpAdm* results based on the Human Origins (HO) panel, following similar filtering and model selection procedures; selected models are also highlighted in yellow. Related to Figure 3.

Table S3. qpAdm admixture modeling results for modern Indonesian populations This table summarizes the results of *qpAdm* analyses used to model the ancestry composition of present-day Indonesian populations based on genome-wide SNP data. (A–C) Results derived from the 1,240K SNP panel: (A) Overview of the basic model parameters, including the selected source populations and outgroup sets used in the admixture framework. (B) Comprehensive list of all tested admixture models, regardless of statistical fit. (C) Subset of models that fulfilled the filtering criteria defined in the Methods section (e.g., acceptable *p-*values, ancestry proportions within logical bounds). Models deemed most reliable for interpretation are highlighted in yellow. Related to Figure 4.

Table S4. qpAdm admixture modeling results for ancient and modern Indonesian populations using Toalean hunter-gatherer as the source Related to Figures 3 and 4.
