# Supplementary material for: Reconstructing the west-east genetic division in Indonesia using ancient genomes
Source: iScience. 2026 May 14;29(6):115974. doi: 10.1016/j.isci.2026.115974 (PMC13196391; doi:10.1016/j.isci.2026.115974)
Supplement: Document S1. Figures S1–S6 [file mmc1.pdf]

iScience, Volume *29*

Supplemental information

Reconstructing the west-east genetic division in Indonesia using ancient genomes

Yu Xu, Ketut Wiradnyana, Hui Zhou, Yinhui Zhao, Xian Wang, Le Tao, Kongyang Zhu, Taufiqurrahman Setiawan, Jianhua Wang, Wen-Jing Lu, Yun Wu, Xueping Ji, Chuan-Chao Wang, and Xiaoming Zhang

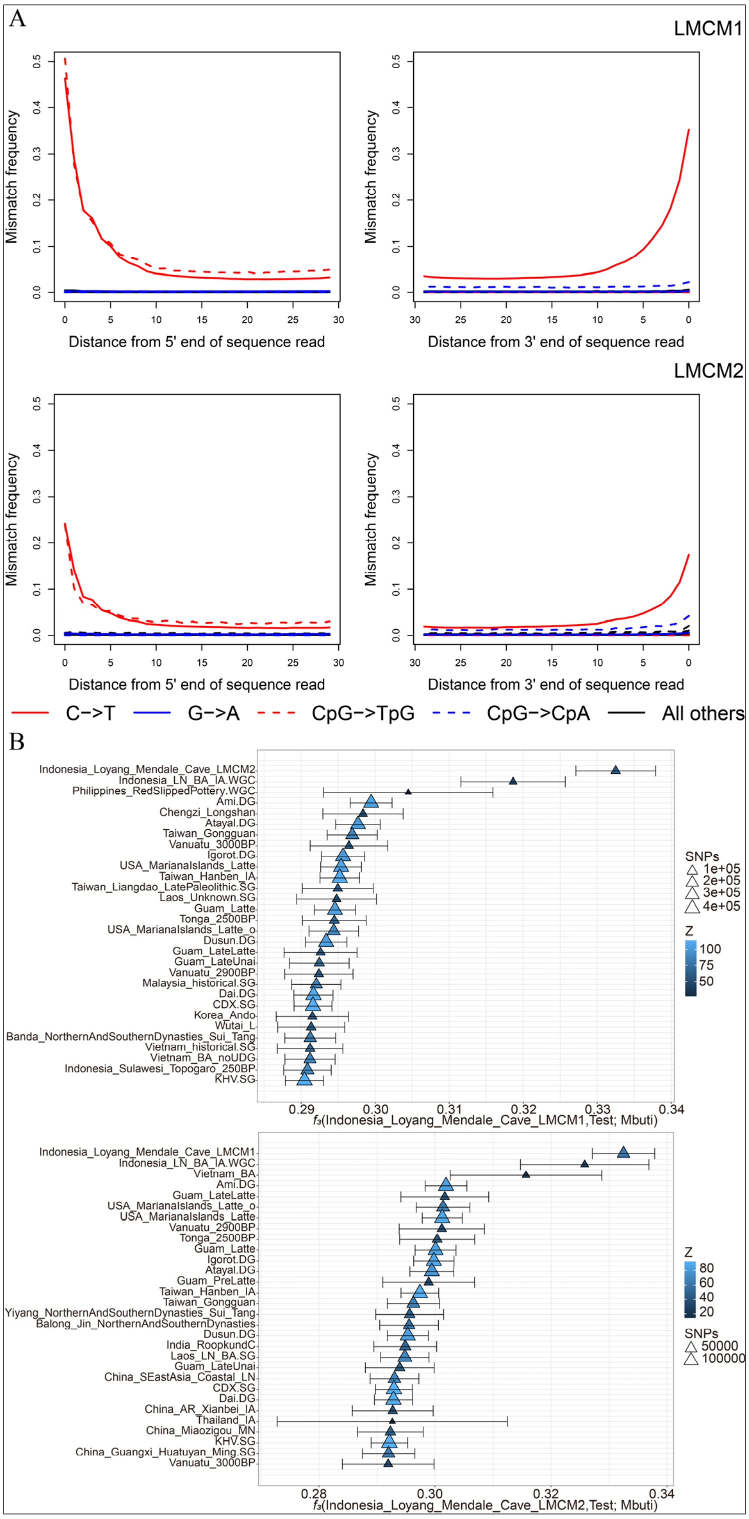

Figure S1. Genetic characterization of newly reported ancient samples. (A) Patterns of post-mortem DNA damage assessed using *PMDtools*, confirming authenticity of ancient DNA. (B) Outgroup-f_3 statistics of f_3(Mbuti; Loyang_Mendale_Cave, X) tests measuring shared genetic drift between the two newly reported individuals and global populations from the 1240K dataset. Related to Table S1. Data are represented as $f_3$values +/− standard errors.

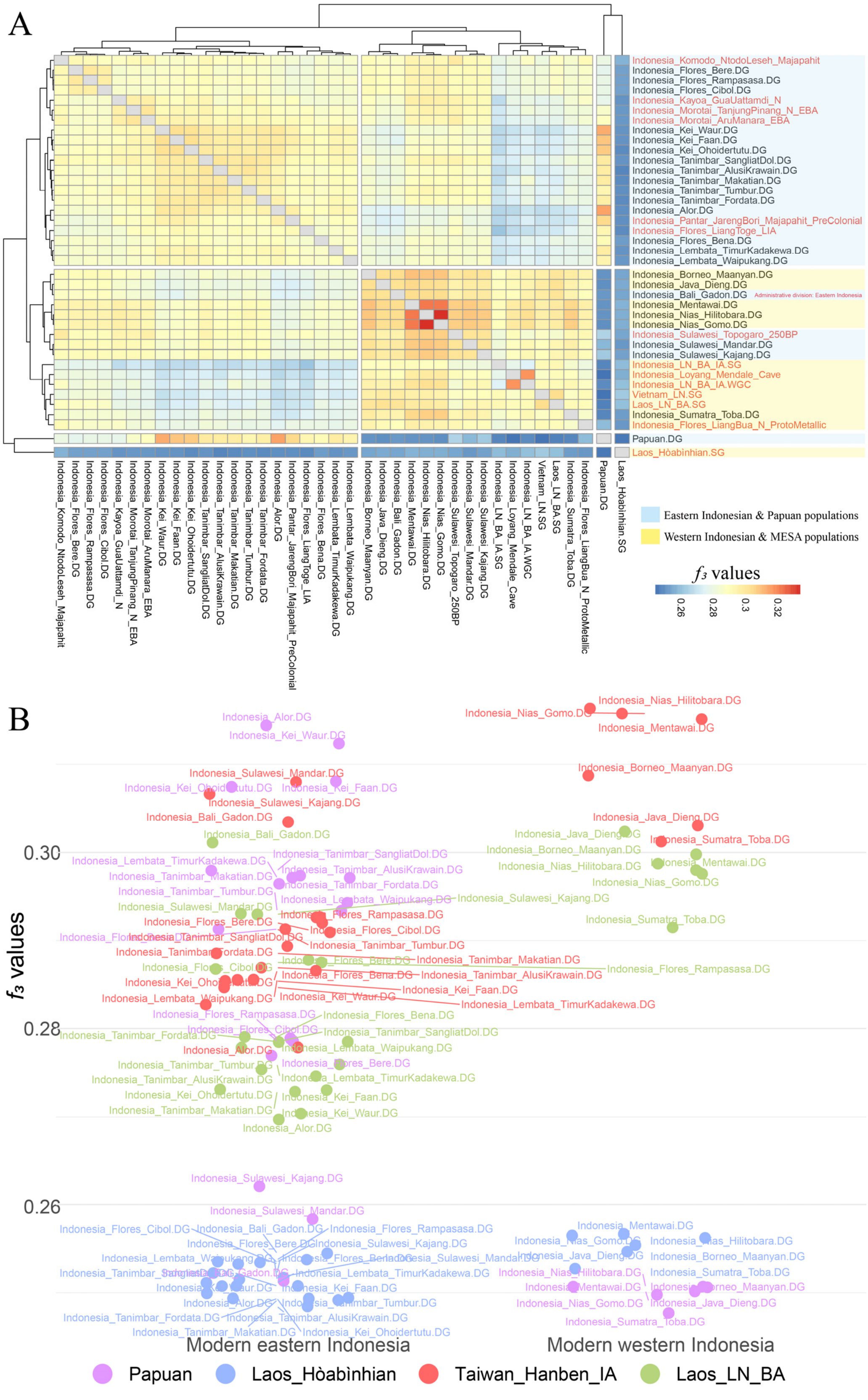

Figure S2. Cluster-based analysis of shared genetic drift among Indonesian populations using outgroup-f_3 statistics. (A) Outgroup-f_3 values of f_3(Mbuti; X, Y)

tests, were computed to assess shared genetic drift between population pairs, with clustering based on genetic similarity. Populations located west of the Wallace Line are shaded in yellow, and those to the east are shaded in blue. Ancient populations are denoted with red labels to facilitate comparison with modern groups. (B) Dot plot summarizing outgroup-f_3 values between modern Western and Eastern Indonesian populations, highlighting differential levels of genetic affinity across the archipelago. These results provide insights into historical gene flow and population structure across the Wallacean biogeographic boundary. Related to Figure 2.

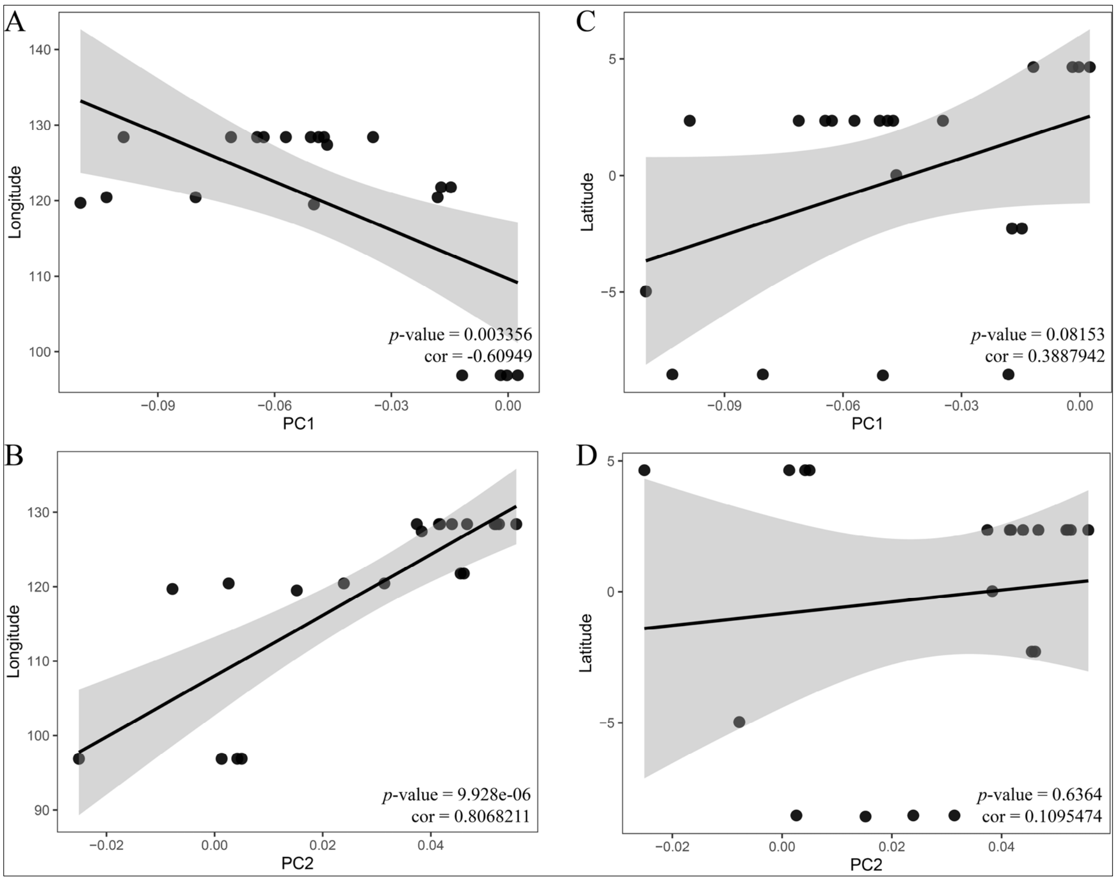

Figure S3. Geographic correlates of genetic structure in ancient Indonesian populations. Correlation analyses between principal component (PC) values and geographic coordinates (latitude and longitude) were performed to assess spatial patterns of genetic variation. Each data point represents an ancient individual, with linear regression models applied to evaluate the extent to which PC1 and PC2 capture longitudinal or latitudinal clines in genetic structure. These results provide supporting evidence for geographic structuring observed in the PCA presented in the main text. Related to Figure 1.

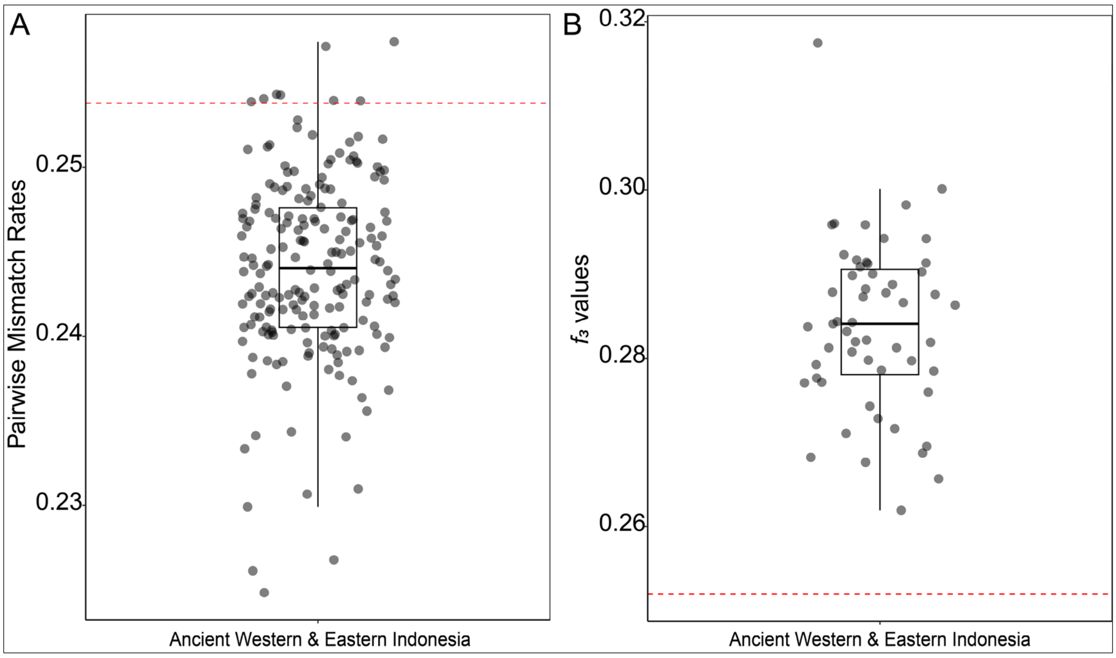

Figure S4. Pairwise genetic divergence and shared ancestry among ancient Indonesian individuals. Dot plot summarizing pairwise mismatch rates and pairwise outgroup-f_3 statistics among ancient Indonesian samples. Mismatch rates were calculated at the individual level to quantify genetic dissimilarity, while outgroup-f_3 values of f_3(Mbuti; X, Y) test were computed at the population level to estimate shared genetic drift. Together, these metrics provide complementary insights into genetic relationships and population structure among ancient individuals. The red line highlights the comparison between *Laos_Hòabìnhian* and *Indonesia_Sulawesi_HG*, emphasizing a notable affinity despite temporal and geographic separation. Related to Figure 2.

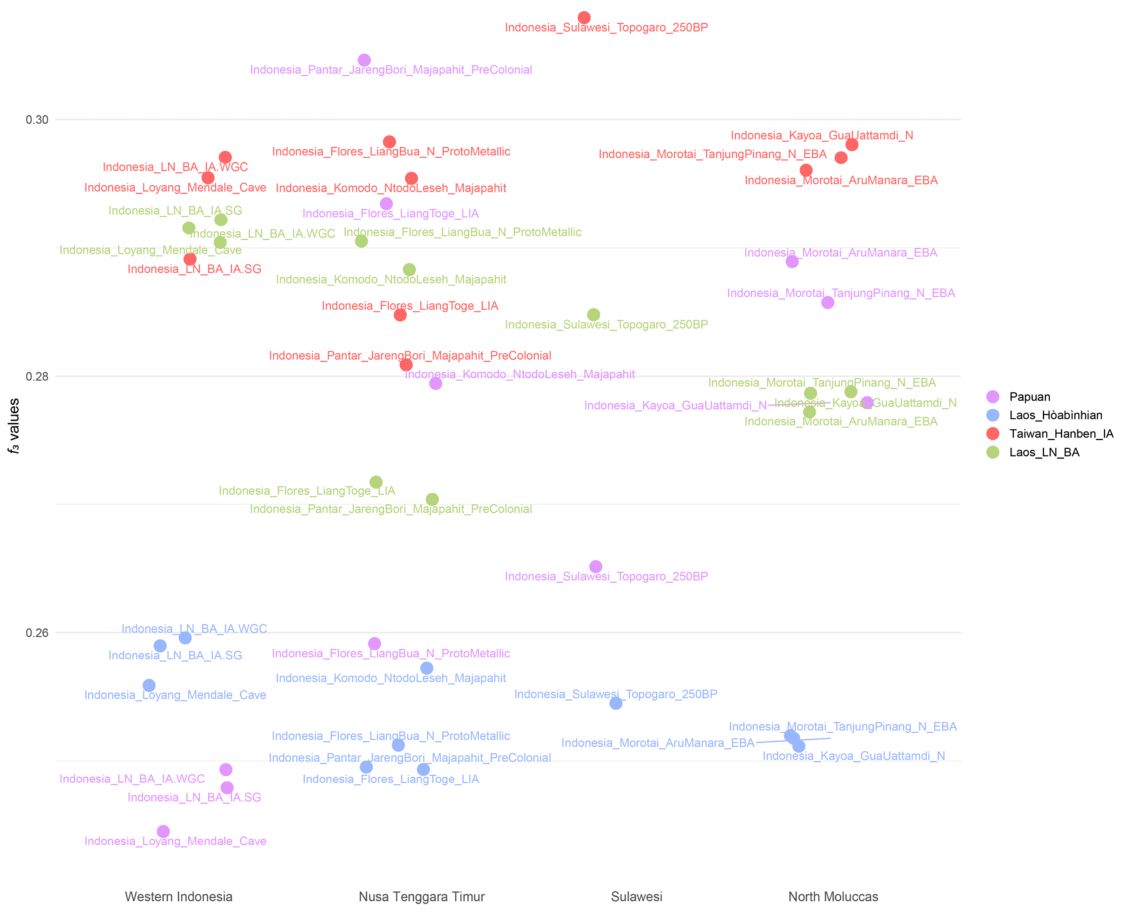

Figure S5. Regional variation in shared genetic drift across Indonesian populations. Dot plot depicting outgroup-f_3 statistics of f_3(Mbuti; target, X) tests for populations sampled from different Indonesian regions. Each point represents the magnitude of shared genetic drift between a target population and a panel of reference groups, enabling comparative analysis of genetic affinities across the archipelago. This regional breakdown highlights patterns of population structure and historical relatedness among Western, Central, and Eastern Indonesian groups. Related to Figure 2.

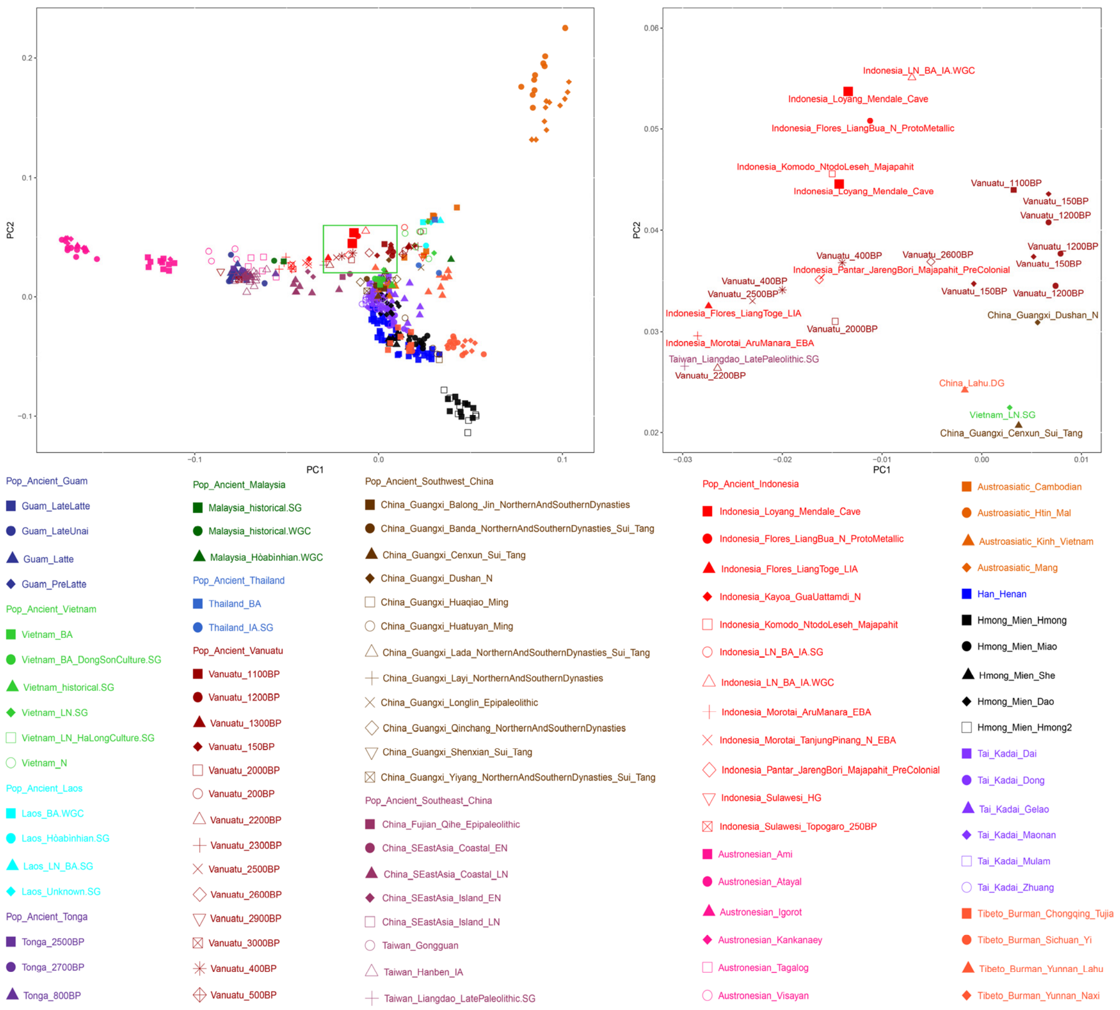

Figure S6. Principal component analysis (PCA) excluding Papuan reference samples. This panel resulted in a reduced genetic differentiation between ancient western and eastern Indonesian populations, leading to a tighter clustering of these groups in the PCA space. Related to Figure 1.
